# Efficacy of Dexmedetomidine versus Ketofol for Sedation of Postoperative Mechanically Ventilated Patients with Obstructive Sleep Apnea

**DOI:** 10.1155/2018/1015054

**Published:** 2018-01-28

**Authors:** Hatem Elmoutaz Mahmoud, Doaa Abou Elkassim Rashwan

**Affiliations:** Anesthesia and Surgical ICU Department, Faculty of Medicine, Beni Suef University, Beni Suef, Egypt

## Abstract

Patients with sleep apnea are prone to postoperative respiratory complications, requiring restriction of sedatives during perioperative care. We performed a prospective randomized study on 24 patients with obstructive sleep apnea (OSA) who underwent elective surgery under general anesthesia. The patients were equally divided into two groups: *Group Dex*: received dexmedetomidine loading dose 1 mcg/kg IV over 10 min followed by infusion of 0.2–0.7 mcg/kg/hr; *Group KFL*: received ketofol as an initial bolus dose 500 mcg/kg IV (ketamine/propofol 1 : 1) and maintenance dose of 5–10 mcg/kg/min. Sedation level (Ramsay sedation score), bispectral index (BIS), duration of mechanical ventilation, surgical intensive care unit (SICU) stay, and mean time to extubation were evaluated. Complications (hypotension, hypertension, bradycardia, postextubation apnea, respiratory depression, and desaturation) and number of patients requiring reintubation were recorded. There was a statistically significant difference between the two groups in BIS at the third hour only (*Group DEX* 63.00 ± 3.542 and *Group KFL* 66.42 ± 4.010, *p* value = 0.036). Duration of mechanical ventilation, SICU stay, and extubation time showed no statistically significant differences. No complications were recorded in both groups. Thus, dexmedetomidine was associated with lesser duration of mechanical ventilation and time to extubation than ketofol, but these differences were not statistically significant.

## 1. Introduction

Obstructive sleep apnea (OSA) is a common condition [1] and is characterized by recurrent episodes of decrease in or cessation of airflow during sleep [2]. This condition causes a decrease in the oxygen level in the blood leading to an increase in the blood pressure and strain on the heart and lungs. The incidence of OSA is nearly 5% and about 9% among surgical patients [3].

Patients with OSA have increased incidence of perioperative complications [4]; they are susceptible to postoperative airway complications and require use of low doses of opioids and sedatives [5]. Sleep apnea is becoming a major concern for intensivists, as these patients need postoperative admission to the intensive care unit (ICU), mechanical ventilation, and sedation [6]. Dexmedetomidine is an *α*2-adrenoreceptor agonist; it has analgesic and sedative properties and is associated with limited respiratory depression [7–9]. Propofol is a sedative-hypnotic agent with rapid onset and short duration of action [10]. Ketamine, an NMDA receptor antagonist, binds to opioid and sigma receptors, leading to dissociative anesthesia [11], amnesia, and analgesia [12]. Its use as a single sedative agent has been limited because it causes emergence reactions [13].

Ketofol, which is a mixture of ketamine and propofol in a single syringe, has been shown to be effective in the operating theater and in day surgeries [14, 15]. It has the advantage of minimizing the respiratory and hemodynamic effects of the constituent drugs [16]. The combined administration of ketamine and propofol has been shown to reduce the dose of propofol needed for sedation [17]. However, the use of ketofol is a new practice for intensivists, and there are limited data on its use as a sedative in the ICU [18].

No previous reports have compared the efficacy of dexmedetomidine and ketofol for postoperative sedation of mechanically ventilated patients with OSA. In this study, we compare the efficacy of dexmedetomidine and ketofol for postoperative sedation of mechanically ventilated patients with OSA in terms of sedation level, duration of mechanical ventilation, time of extubation, duration of surgical intensive care unit (SICU) stay, and occurrence of complications.

## 2. Materials and Methods

This single-center randomized study was conducted in the SICU of Benisuef University Hospital. We obtained approval from the ethics committee of the institution (The FM-BSU REC). The study was registered at ISRCTN (trial registration number: ISRCTN56992547).

After obtaining consent, 24 patients diagnosed with OSA, who underwent elective surgeries under general anesthesia from May 2016 to April 2017, were included. These patients were admitted to the SICU, and were intubated, ventilated, and sedated according to the protocol followed in our department, as they may develop postoperative respiratory depression and/or obstruction and need reintubation.

### 2.1. Inclusion Criteria

The study included adult patients (18–50 years) with OSA requiring postoperative short-term sedation and mechanical ventilation (less than 12 hours).

### 2.2. Exclusion Criteria

1. Requirement for prolonged sedation and mechanical ventilation (more than 12 hours)
2. Epilepsy
3. Known allergies to the drugs being studied
4. Severe hepatic, renal, or central nervous system involvement, significant cardiac diseases, or arrhythmias
5. Pregnancy
6. Intake of other sedatives and anticonvulsant drugs

Intraoperative analgesia was maintained in all patients with fentanyl 1 mcg/kg, followed by infusion of 1-2 mcg/kg/h; the administration was ceased at the end of the operation.

On arrival to the SICU, the patients were connected to the mechanical ventilator; complete monitoring was performed using ECG, pulse oximetry, noninvasive and invasive arterial blood pressure measurement, and capnography. Bispectral index (BIS) electrodes were applied on the forehead. A baseline 12-lead ECG, chest radiograph, ABGs, and CBC were obtained, and biochemical tests were performed.

Patients were randomly allocated into two groups by a sealed opaque envelop technique: *Group Dex* comprised twelve patients receiving a loading dose infusion of dexmedetomidine (Precedex, Abbot Laboratories Inc., Abbot Park, IL, USA; 2 ml, 200 mcg vial, 100 mcg/ml) 1 mcg/kg IV over 10 min, followed by infusion of 0.2–0.7 mcg/kg/hr [19]. *Group KFL* comprised twelve patients receiving ketofol as an initial bolus dose 500 mcg/kg IV (ketamine/propofol 1 : 1; ketamine 8 mg/ml and propofol 8 mg/ml, by mixing 40 ml propofol 1% (10 mg/ml)) with 8 ml ketamine (50 mg/ml) and 2 ml dextrose 5% (each ml of aliquot contained 8 mg propofol and 8 mg ketamine), followed by infusion of 5–10 mcg/kg/min [18].

The degree of sedation was measured hourly using the Ramsay sedation score (RSS). In both groups, the target was to achieve and maintain RSS of 4 or 5.

### 2.3. Ramsay Sedation Scale

Sedation level description is as follows:

1. Patient is anxious and agitated or restless, or both.
2. Patient is cooperative, oriented, and tranquil.
3. Patient responds to commands only.
4. Patient exhibits brisk response to light glabellar tap or loud auditory stimulus.
5. Patient exhibits a sluggish response.
6. Patient exhibits no response [20].

When the patients fulfilled the criteria for weaning and extubation [21], mechanical ventilation was discontinued and extubation was performed. We collected the following data: (1) demographic data: age, sex, body mass index, and types of surgeries; (2) vital signs: heart rate, invasive mean arterial blood pressure, SpO_2_, and end-tidal CO_2_, which were continuously monitored and recorded at baseline (after admission to the SICU), at 1 hour and 3 hours after the start of sedation, and then every 3 hours; (3) sedation level: RSS was recorded at baseline, at 1 hour and 3 hours after the start of sedation, and then every three hours; (4) BIS was recorded at baseline, at 1 hour and three hours after the start of sedation, and then every three hours; (5) duration of mechanical ventilation, and stay in the SICU (hours) (secondary outcome); (6) mean time to extubation (the time of discontinuation of sedative to extubation in minutes) (primary outcome); (7) behavioral pain scale for pain assessment recorded at baseline, at 1 hour, and 3 hours after the start of sedation, and then every 3 hours (Table 1) [22]; (8) complications including hypotension (systolic blood pressure less than 90 mmHg), hypertension (systolic blood pressure more than 170 mmHg), and bradycardia (heart rate less than 50 b/minute) [18].

Additionally, the number of patients who required reintubation and those who had postextubation respiratory depression, apnea, and desaturation was recorded.

### 2.4. Statistical Analysis

After a pilot study with three patients in each group, the mean ± SD of extubation time in dexmedetomidine treated group was 32.3 ± 2.1 minutes, while in ketofol group was 39 ± 2.2 minutes. Accordingly, we calculated that the minimum proper sample size was 10 participants in each arm to be able to detect a real difference of 13.2 minutes with 95% power at *α* = 0.05 level using Student's *t*-test for independent samples. We increased the number to 12 patient in each group in case of drop of any case. Sample size calculation was done using Stats Direct statistical software version 2.7.2 for MS Windows, Stats Direct Ltd., Cheshire, UK. We performed analysis using computer program IBM SPSS (Statistical Package for the Social Science; IBM Corp, Armonk, NY, USA) release 22 for Microsoft Windows. Data were statistically described in terms of mean ± standard deviation (±SD), median and range, or frequencies (number of cases) and percentages when appropriate. Comparison of numerical variables between the study groups was done using the Mann–Whitney *U* test for independent samples. For comparing categorical data, the chi-square (*χ*^2^) test was performed. The exact test was used instead when the expected frequency is less than 5. *p* values less than 0.05 were considered statistically significant.

## 3. Results

**Figure 1:**
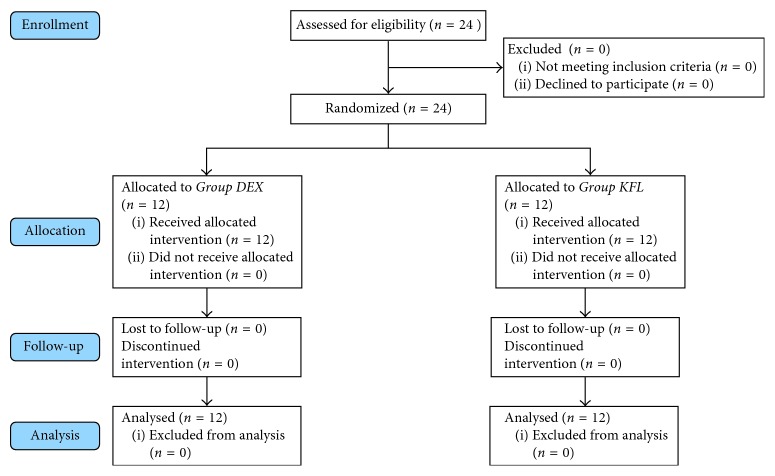
Consort flow participant diagram.

**Figure 2:**
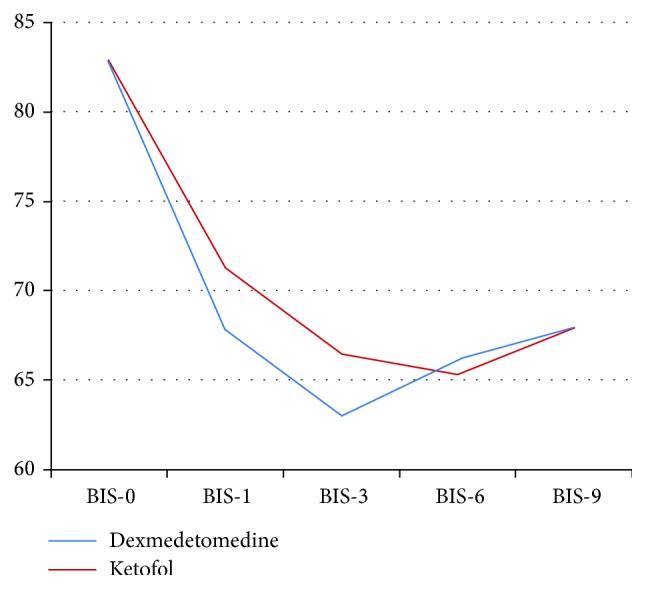
Mean BIS between the study groups over the study period.

**Figure 3:**
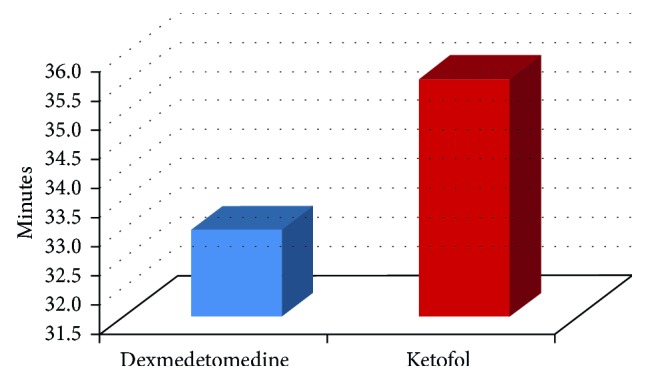
Mean extubation time (min) between the study groups.

**Figure 4:**
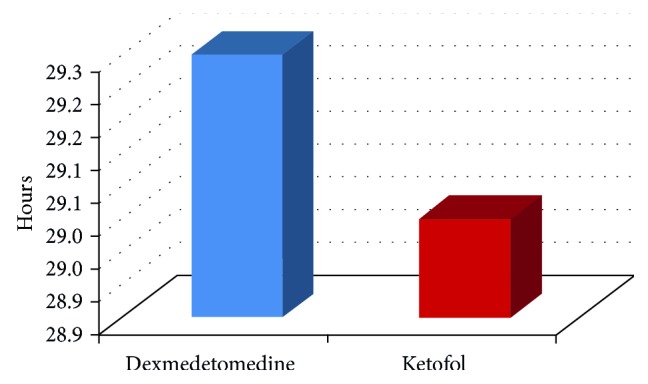
Mean SICU stay (hours) between the study groups.

We included 24 patients in this study. All cases completed the study (Figure 1). No statistical significant differences in the demographic data and types of surgeries between the two groups (Table 2). The heart rate was statistically significantly lower in *Group DEX* than *Group KFL* at 1, 3, 6, 9, 12, and 18 hours (Table 3). The mean arterial blood pressure was statistically significantly lower in *Group DEX* than *Group KFL* at 15, 18, and 21 hours (Table 4). No statistical significant differences between the two groups in SpO_2_ and end-tidal CO_2_. No statistical significant differences in Ramsay sedation score between the two groups (Table 5). There was a statistical significant difference between the two groups in BIS at 3 hours only, it was 63.00 ± 3.542 in *Group DEX* and 66.42 ± 4.010 in *Group KFL* (*p* value = 0.036) (Table 6, Figure 2). No statistical significant differences in the behavioral pain scale between the two groups (Table 7). The duration of mechanical ventilation, extubation time (Figure 3), and length of the SICU stay (Figure 4) was lower in *Group DEX* than *Group KFL* without statistically significant difference (Table 8). No hypotension, hypertension, bradycardia, postextubation respiratory depression, apnea, or desaturation recorded. No patients required reintubation in both groups.

## 4. Discussion

The results of the present study showed that both dexmedetomidine and ketofol were effective for sedation of postoperative mechanically ventilated patients with obstructive sleep apnea and provided hemodynamic stability without complications.

Obstructive sleep apnea is characterized by periodic, partial, or complete obstruction of the upper airway, resulting in the disruption of sleep and hypoxemia [23].

Patients with OSA are prone to postoperative respiratory problems after general anesthesia [24, 25].

Sedation and analgesia used in critical care units provide patients with comfort and safety [26].

Dexmedetomidine, an alpha-2 agonist, may reduce the duration of mechanical ventilation [27]; it is a useful adjunct in surgical patients with OSA [5], as it has analgesic and sedative properties and limited respiratory depression. It is useful in patients with OSA undergoing surgeries associated with significant postoperative pain [28, 29].

Propofol and ketamine, when used in combination, provided effective sedation for spinal anesthesia and cardiovascular procedures [30]; it has been used for sedation in awake craniotomy and maintained hemodynamic and respiratory stability and is associated with rapid recovery profile [31].

Xu et al. [32] in their study compared propofol with dexmedetomidine for sedation of adults who were mechanically ventilated after uvulopalatopharyngoplasty in the PACU, and the bispectral index values were significantly lower in the dexmedetomidine group than in the propofol group. The times to spontaneous breathing, awaking, and extubation were shorter in the dexmedetomidine group. They concluded that dexmedetomidine is an effective sedative for mechanically ventilated adults following uvulopalatopharyngoplasty.

Eremenko and Chemova [33] compared the efficacy of dexmedetomidine and propofol for short-term sedation and analgesia after cardiac surgery; they reported no significant differences in the duration of mechanical ventilation or rate of awakening between the groups. Dexmedetomidine provides analgesic effect and shortens the duration of ICU stay. Bradycardia was observed more in dexmedetomidine while arterial hypotension in the propofol group.

Paliwal et al. [19] showed a statistically significant lower heart rate in dexmedetomidine group; the decrease in mean arterial pressure was more in the propofol group.

A study by Srivastava et al. [34] reported that dexmedetomidine maintained hemodynamic stability compared to propofol and midazolam for sedation of neurosurgical mechanically ventilated patients.

Elbaradei et al. [35] showed that dexmedetomidine and propofol are safe sedatives for postoperative short-term ventilation and that dexmedetomidine resulted in lower heart rates than propofol.

In our study, ketofol was used for short-term sedation with no complications reported; similarly, Hamimy et al. [18]. concluded that ketofol infusion provided adequate short-term sedation (less than 24 h) in mechanically ventilated patients with rapid recovery and no significant complications.

## 5. Conclusion

Dexmedetomidine was associated with lower duration of mechanical ventilation and less time for extubation than ketofol for sedation of postoperative mechanically ventilated patients with obstructive sleep apnea, but these differences were not statistically significant. Both provided hemodynamic stability without complications.

## Figures and Tables

**Table 1:** Behavioral pain scale for pain assessment.

Item	Description	Score
Facial expression	Relaxed	1
	Partially tightened (e.g., brow lowering)	2
	Fully tightened (e.g., eyelid closing)	3
	Grimacing	4

Upper limbs	No movement	1
	Partially bent	2
	Fully bent with finger flexion	3
	Permanently retracted	4

Compliance with ventilation	Tolerating movement	1
	Coughing but tolerating ventilation for most of the time	2
	Fighting ventilator	3
	Unable to control ventilation	4

**Table 2:** Demographic data and surgical procedures in both groups.

Variable	*Group KFL* (*n* = 12)	*Group DEX* (*n* = 12)	*p* value
Age (years)	36.58 ± 10.850	34.17 ± 8.111	0.644
BMI (kg/m^2^)	48.75 ± 9.343	44.58 ± 10.917	0.452
Sex (M/F)	6/6	5/7	1.000
Type of surgery (laparoscopic gastric sleeve/uvulopalatoplasty/lumbar disc fixation)	6/5/1	5/4/3	—

Data are presented as mean ± SD. *p* values ≤ 0.05 are considered statistically significant.

**Table 3:** Heart rate (Bpm).

Variable			*p* value
Time (hr)	*Group KFL* (*n* = 12)	*Group DEX* (*n* = 12)	
0	88.42 ± 5.125	87.75 ± 4.224	0.580
1	80.67 ± 5.774	73.00 ± 4.390	0.003^∗^
3	77.25 ± 4.137	66.00 ± 4.134	0.000^∗^
6	80.42 ± 2.778	71.67 ± 9.74	0.013^∗^
9	83.08 ± 4.055	76.67 ± 3.846	0.001^∗^
12	86.42 ± 4.274	82.42 ± 4.776	0.049^∗^
15	86.08 ± 2.875	84.17 ± 4.726	0.368
18	84.33 ± 4.418	79.08 ± 5.334	0.026^∗^
21	82.42 ± 4.295	78.83 ± 5.638	0.181
24	82.58 ± 4.055	83.25 ± 6.426	0.931
27	83.00 ± 4.090	84.08 ± 6.302	0.469
30	84.83 ± 4.196	82.50 ± 5.854	0.311

Data are presented as mean ± SD. ^∗^*p* values ≤ 0.05 are considered statistically significant. Bpm = beat per minute.

**Table 4:** Mean arterial blood pressure (mmHg).

Variable	MAP		*p* value
Time (hr)	*Group KFL* (*n* = 12)	*Group DEX* (*n* = 12)	
0	101.58 ± 13.714	101.25 ± 10.922	0.977
1	96.75 ± 6.524	90.33 ± 13.412	0.202
3	92.58 ± 6.802	89.08 ± 10.104	0.311
6	87.17 ± 3.857	83.92 ± 10.361	0.642
9	85.50 ± 6.488	84.83 ± 10.035	0.794
12	86.17 ± 3.512	83.25 ± 7.736	0.415
15	84.58 ± 6.317	78.17 ± 7.396	0.037^∗^
18	84.25 ± 5.379	79.08 ± 6.082	0.046^∗^
21	100.92 ± 13.358	92.58 ± 4.100	0.009^∗^
24	95.50 ± 9.060	92.00 ± 7.160	0.349
27	94.00 ± 7.032	94.25 ± 9.910	0.663
30	92.33 ± 4.119	94.17 ± 7.779	0.448

Data are presented as mean ± SD. ^∗^*p* values ≤ 0.05 are considered statistically significant. MAP = mean arterial blood pressure.

**Table 5:** Ramsay sedation score.

Time (hrs)	*Group KFL* (*n* = 12)	*Group DEX* (*n* = 12)	*p* value
0	1(1-2)	1 (1-2)	1.000
1	4 (3–5)	4 (4–5)	0.244
3	4 (4–5)	4 (4–5)	1.000
6	3 (2–4)	4 (2–4)	0.126
9	2 (2-3)	2 (2-3)	0.680
12	2 (1-2)	2 (2-3)	1.000

Data are presented as median and range. *p* values  ≤ 0.05 are considered statistically significant.

**Table 6:** Bispectral index.

Time (hours)	*Group KFL* (*n* = 12)	*Group DEX* (*n* = 12)	*p* value
0	82.83 ± 3.243	82.75 ± 2.896	0.907
1	71.25 ± 4.827	67.83 ± 6.013	0.156
3	66.42 ± 4.010	63.00 ± 3.542	0.036
6	65.33 ± 2.964	66.17 ± 3.589	0.579
9	67.92 ± 4.757	68.00 ± 6.310	1.000

Data are presented as mean ± SD. *p* values ≤ 0.05 are considered statistically significant.

**Table 7:** Behavioral pain scale.

	*Group KFL* (*n* = 12)	*Group DEX* (*n* = 12)	*p* value
1	1 (1–3)	1 (1–3)	0.156
3	1 (1-2)	1 (1-2)	0.950
6	(1-2)	1 (1-2)	0.317
9	1 (1-2)	1 (1-2)	1.000

Data are presented as median and range. *p* values ≤ 0.05 are considered statistically significant.

**Table 8:** Extubation time, duration of mechanical ventilation, and SICU stay.

Variable	*Group KFL* (*n* = 12)	*Group DEX* (*n* = 12)	*p* value
Extubation time (minutes)	35.58 ± 3.895	33.00 ± 3.384	0.105
Duration of mechanical ventilation (hr)	7.88 ± 3.328	7.58 ± 3.183	0.838
Stay in the SICU (hr)	29.00 ± 2.954	29.25 ± 3.415	0.708

Data are presented as mean ± SD. *p* values ≤ 0.05 are considered statistically significant.

## Additional Points

Small sample size is the limitation of the study. Future studies are recommended using larger sample size and longer duration of mechanical ventilation.
